# Acceptance of interprofessional learning between medical and pharmacy students in a prescribing skills training workshop: pre-post intervention study

**DOI:** 10.1186/s12909-019-1525-y

**Published:** 2019-04-05

**Authors:** Siew Siang Chua, Pauline Siew Mei Lai, Si Mui Sim, Choo Hock Tan, Chan Choong Foong

**Affiliations:** 1 Department of Pharmacy, Faculty of Medicine, University of Malaya, Kuala Lumpur, Malaysia; 2 Department of Primary Care Medicine, Faculty of Medicine, University of Malaya, Kuala Lumpur, Malaysia; 3 Department of Pharmacology, Faculty of Medicine, University of Malaya, Kuala Lumpur, Malaysia; 4 Medical Education and Research Development Unit (MERDU), Faculty of Medicine, University of Malaya, Kuala Lumpur, Malaysia; 5 School of Pharmacy, Faculty of Health and Medical Sciences, Taylor’s University, Lakeside Campus, Subang Jaya, Selangor Malaysia

**Keywords:** Interprofessional learning, Medical student, Pharmacy students, Prescribing skills training, SAIL-10, Validation

## Abstract

**Background:**

The success of interprofessional collaboration in healthcare services requires a paradigm shift in the training of future health profession practitioners. This study aimed to develop and validate an instrument to measure Student Acceptance of Interprofessional Learning (SAIL) in Malaysia, and to assess this attribute among medical and pharmacy students using a prescribing skills training workshop.

**Methods:**

The study consisted of two phases. In Phase 1, a 10-item instrument (SAIL-10) was developed and tested on a cohort of medical and pharmacy students who attended the workshop. In Phase 2, different cohorts of medical and pharmacy students completed SAIL-10 before and after participating in the workshop.

**Results:**

Factor analysis showed that SAIL-10 has two domains: “facilitators of interprofessional learning” and “acceptance to learning in groups”. The overall SAIL-10 and the two domains have adequate internal consistency and stable reliability. The total score and scores for the two domains were significantly higher after students attended the prescribing skills workshop.

**Conclusions:**

This study produced a valid and reliable instrument, SAIL-10 which was used to demonstrate that the prescribing skills workshop, where medical and pharmacy students were placed in an authentic context, was a promising activity to promote interprofessional learning among future healthcare professionals.

## Background

Healthcare has become increasingly patient-centered, where interdisciplinary teams revolve to provide patients with the best integrated health services. This is particularly important for patients with complex and chronic health needs that are best addressed by a collaborative team of healthcare professionals such as doctors and pharmacists [1, 2]. Management of medications is enhanced when doctors and pharmacists work as a team to diagnose, prescribe, supply and counsel on the use of medications [3]. Failure of such collaboration (for example, miscommunication between healthcare providers) may lead to inadvertent harm to the patients [4].

Medical and pharmacy training programmes at most universities are structured to accommodate their specific disciplines [5], resulting in a lack of integrated interprofessional training opportunities. The interprofessional “gap” is often broadened further by a traditional healthcare hierarchy and an old paradigm, where doctors are perceived to have an ascendant role over other healthcare professionals, and also as the core or principal healer [6]. A lack of exposure to and an inadequate interaction between medical and pharmacy students has resulted in the lack of knowledge, trust and confidence in the roles of other healthcare providers and on what, or how they should work together after graduation. A study conducted in Canada reported that medical graduates agreed that doctors and pharmacists have equal responsibilities in knowing the contraindications of a medication, and in assessing patients’ adherence to their medications. Medical graduates also expressed their willingness to work with pharmacists, but were reserved on how to advise patients on lifestyle changes [7]. This is also a relevant concern for countries which are without, or are moving towards, dispensing separation such as Malaysia. Doctors and pharmacists should learn about their respective professional responsibilities, and how to best work together as a team, to fully utilise each other’s skills and knowledge, to ensure optimal patient care.

The success of interprofessional collaboration in healthcare services requires a paradigm shift in the training of future healthcare professionals. An approach, called “interprofessional learning” (IPL), which conceptually means “when students from two or more professions learn about, from and with each other to enable effective collaboration and improve health outcomes” [8], is perhaps the best strategy that should be initiated early in the training of healthcare professionals. Several studies have concluded that IPL at the undergraduate level should be designed to increase students’ awareness of the roles and responsibilities of other professions, increase the understanding of teamwork between healthcare professionals, as well as to increase the understanding of the contributions made by different professions. This is in addition to understanding their own professional identity, obligations and limitations [9–12].

Despite global advocacy at universities, the implementation of IPL in the early stages of professional training is associated with various challenges. These include difficulties in incorporating IPL into the current time table, the different length of the medical and pharmacy programmes, the distinct assessment methods of the various programmes, a lack of commitment and support from administrators, difficulties in coordinating and limited resources, and (perhaps the most intrinsically important) the attitudinal factor in students [13–17]. The very first step in implementing IPL, that is the introduction of the concept and the programme to students, is the most crucial step so that students from different backgrounds and courses are willing to embrace and be committed to learning together.

Several instruments have been developed specifically to evaluate students’ attitudes or readiness towards IPL: the Interdisciplinary Education Perception Scale (IEPS) [18], the Interprofessional Attitudes Questionnaire [19], and the Readiness for Inter-Professional Learning Scale (RIPLS) [13]; among which the RIPLS [20–24] and the IEPS [25, 26] appeared to be the most widely used instruments. RIPLS is useful prior to the implementation of IPL activities, as it gauges the readiness of healthcare students for IPL. However, this instrument has recently received criticism for its psychometric properties and factor structures [27]. IEPS, an outcome-based instrument, measures the effect of IPL on students’ attitudes regarding interprofessional collaboration. The attitudes of students towards IPL have been assessed in several countries such as the United Kingdom, Ireland, Canada, New Zealand and Malaysia [14, 15, 20, 23, 24, 28].

In summary, there are existing instruments, but no single instrument would be feasible to accommodate the unique education and practice environment of each country (Gillian et al., 2011). Thus far, only a partial validation on RIPLS had been performed in Malaysia [24], and no instrument has been developed and validated to assess IPL between medical and pharmacy students in Malaysia. Therefore, the aim of the present study was to develop and validate an instrument to measure student acceptance of IPL in Malaysia, and to assess this attribute among medical and pharmacy students using a prescribing skills training workshop.

## Methods

This study consisted of two phases. Phase 1 described the development and validation of an instrument used for measuring student acceptance of interprofessional learning (SAIL). Phase 2 described the use of the SAIL-10 instrument to assess the acceptance of medical and pharmacy students on IPL using a prescribing skills training workshop. Ethics approval was granted by the Medical Research Ethics Committee, University Malaya Medical Centre (MREC ID NO: 20168–2647).

### Phase 1: The development and validation of the student acceptance of Interprofessional learning (SAIL) instrument

#### Face and content validity

The SAIL instrument was developed based on literature review, and the experience of an expert panel (consisting of two pharmacists, a pharmacologist and a medical doctor, who were involved in the teaching of medical or pharmacy students), which confirmed the content validity of the instrument. The initial SAIL instrument consisted of 12 items (SAIL-12), with a 6 point Likert-like scale response, where 1 represented “Strongly disagree” and 6 represented “Strongly agree”.

#### Pilot study

The SAIL-12 instrument was piloted on 108 students (64 medical and 44 pharmacy students) who attended a prescribing skills training workshop. Students were asked to answer SAIL-12 after they attended the prescribing skills workshop and to evaluate verbally if the items were difficult for them to comprehend. The Cronbach’s alpha value of SAIL-12 was 0.802, which indicated good internal consistency. All items had corrected item-total correlation of more than 0.2 [29]. However, we decided to exclude two items from the SAIL-12 instrument (items no. 5 and 11), as item 5 was very similar to item 10, and item 11 only had a corrected item-total correlation of 0.228. When these two items were excluded, the Cronbach’s alpha value for the 10-item SAIL instrument increased slightly to 0.806.

#### SAIL-10 instrument

The SAIL-10 instrument consists of 10 items, with a 6 point Likert-like scale response, where 1 represented “Strongly disagree” and 6 represented “Strongly agree”. The individual item score was converted to range from 0 to 5, and the total score was converted to percentage. We hypothesized that our instrument would have one domain which assessed the IPL between medical and pharmacy students.

#### Study population

Final year medical and pharmacy students from the Faculty of Medicine, University of Malaya, Kuala Lumpur, who attended the prescribing skills workshop were recruited for this study. Sample size was calculated based on the number of items in SAIL-10 to participant ratio. Recommendations range from 1:5 [30] to 1:10 [31]. Since SAIL-10 has 10 items, hence the minimum number of participants required should be between 50 and 100.

#### Intervention: the prescribing skills workshop

**Fig. 1:**
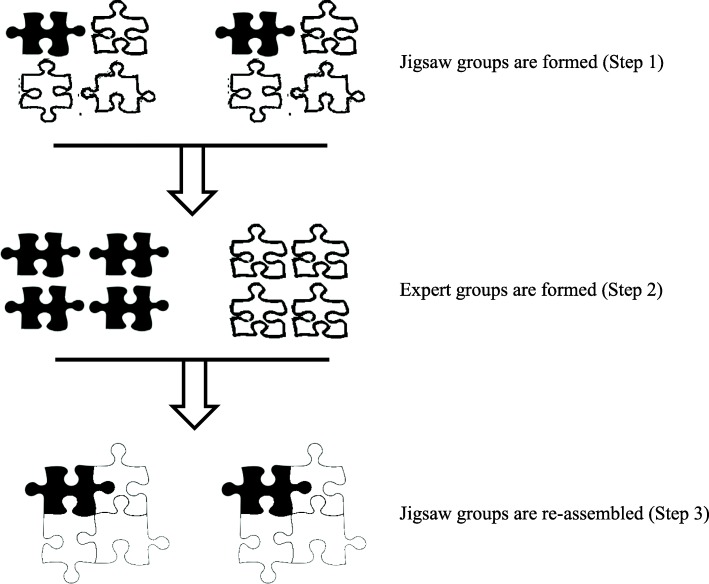
General steps in using “Jigsaw” technique

This workshop was conducted using the “Jigsaw” learning technique [32]. It was divided into four sessions: introductory (30 min), “Expert” group (60 min), “Jigsaw” group (100 min) and debriefing sessions (20 min) [33]. Training of students on this learning technique was essential. The introductory session exposed the medical and pharmacy students to common dilemmas in prescribing and briefed the students on the learning objectives and procedures of the “Jigsaw” learning technique. “Jigsaw” learning technique is a cooperative learning technique, where every student can be thought of as a segment of the jigsaw puzzle (i.e., learning content of the day) and each student contributes to complete the puzzle (Fig. 1) [32]. In a “Jigsaw” classroom, learning content of the day is divided into a number of segments. Students will form “Jigsaw” groups (also called Home Teams in this study). In each “Jigsaw” group there is at least one student assigned as a particular “segment”, and students of the same segment from different “Jigsaw” groups will convene in a separate “Expert” group to master the learning content corresponding to the particular segment. Learning activities in “Expert” group are usually facilitated by capable peers and instructors or through on-the-spot and pre-reading materials. Lastly, students are re-assembled into their respective “Jigsaw” groups. Student experts of each segment are responsible to share their knowledge acquired from the “Expert” group to the rest of the “Jigsaw” group members.

In this study, each workshop involved 32 to 36 final year medical students and 16 to18 final year pharmacy students. The students were divided into two “Jigsaw” groups (also known as “Home Teams” in this study) which consisted of 24 to 27 students (in a ratio of approximately 2 medical to 1 pharmacy student). The students were further assigned randomly to six “Expert” groups. Each group attended one of the six “Expert” stations which were arranged to cover the steps involved in the process of treating a patient with personal selection of drugs (called P-drugs), as recommended by the World Health Organisation (WHO) Guide to Good Prescribing [34]. The learning at each “Expert” station was facilitated by a pharmacologist, pharmacist or medical doctor.

After the “Expert” station learning activities, both medical and pharmacy students reassembled into the two “Home Teams” or “Jigsaw” groups. Each “Jigsaw” group was further divided into three sub-groups, consisting of participants from the six different “Expert” groups, so that students could teach each other what they had learnt at their respective “Expert” stations. Each “Jigsaw” sub-group was required to discuss and solve a different series of six case scenarios, each scenario illustrating how to carry out one of the six steps in the process of drug treatment. One of the sub-group members would present the case to the whole “Jigsaw” group. Two facilitators managed a “Jigsaw” group concurrently and they would challenge the presenters to provide rationales for their given answers or inquired about possible alternatives. Prompt feedback was given by the facilitators to close each case presentation.

During both “Expert” and “Jigsaw” group sessions, the facilitators observed and encouraged interaction and communication between the medical and pharmacy students as well as between student experts and student learners (in the “Jigsaw” groups).

#### Data collection

All students who attended the prescribing skills workshop were requested to fill in the SAIL-10 instrument after they had completed the workshop. Students usually took approximately 10–15 min to complete SAIL-10. The researcher checked the questionnaire to ensure that all questions were answered. After 4 weeks, SAIL-10 was re-administered to the same students.

#### Statistical analyses

All data were entered and analysed using IBM SPSS Statistics for Windows, Version 20.0. (IBM Corp., Armonk, NY). Descriptive statistics were presented as percentage and frequencies for all categorical data, while median and interquartile range (IQR) were calculated for continuous variables. A continuous variable is defined as a variable that has an infinite number of possible values (such as the SAIL score).

#### Validity

Flesch reading ease was calculated to assess the reading comprehension level of SAIL-10. This was calculated based on the average number of syllables per word and words per sentence. The higher the score, the easier it is to understand the document. An average document should have a score between 60 and 70 [35].

The dimensionality of the SAIL-10 instrument was analysed using exploratory factor analysis, with principal component as the extraction method and Varimax with Kaiser normalisation as the rotation method. An eigenvalue > 1 on the Scree plot indicates that more than one component exist in the instrument [36]. Factor loadings should be > 0.4. The Kaiser-Meyer Olkin (KMO) and Bartlett’s Test measure of sampling adequacy were used to examine the appropriateness of factor analysis. KMO should be more than 0.7, and the *p*-value of the Bartlett’s Test measure should be < 0.05 [29].

The Mann-Whitney U-test was used to determine whether SAIL-10 was able to discriminate between medical and pharmacy students’ acceptance toward IPL. A *p*-value < 0.05 was considered as statistically significant.

#### Reliability

Internal consistency was assessed using Cronbach’s alpha coefficient to determine whether all items in a multi-item scale measured the same concept. This was calculated for the entire instrument, and for each subscale. An instrument with Cronbach’s alpha value of ≥ 0.5 is considered to have adequate internal consistency [29, 37]. The corrected item-total correlation was also performed. Corrected item-total correlations were first used to identify items which did not agree well with other items in the questionnaire. Item-total correlations should be > 0.2 to be considered acceptable [37]. The effect of removing an item on the Cronbach’s α was also determined.

Spearman’s rho correlation was used to assess the stability of our instrument: little or no correlation (0–0.25), fair correlation (0.25–0.5), moderate to good correlation (0.5–0.75) and very good to excellent correlation (> 0.75) [38].

### Phase 2: Assessment of student acceptance of interprofessional learning

The assessment of final year medical and pharmacy students’ acceptance of IPL was conducted in the same setting as Phase 1 of the study, from October 2013 to April 2015. Two cohorts of medical and pharmacy students went through the same prescribing skills workshop as those who were in the validation study mentioned above. All students who attended the prescribing skills workshop were requested to fill in SAIL-10 before and after they had attended the workshop.

#### Statistical analyses

All data were entered and analysed using IBM SPSS Statistics for Windows, Version 20.0. (IBM Corp., Armonk, NY) as described for Phase 1 of the study. Descriptive statistics were presented as percentage and frequencies for all categorical data, while median and interquartile range (IQR) were calculated for continuous variables. Since normality of data could not be assumed, the Mann-Whitney U-test was used to determine any difference in SAIL-10 scores between medical and pharmacy students. In addition, the effect of the prescribing skills workshop on the acceptance of IPL was analysed using the Wilcoxon signed ranks test. A *p*-value < 0.05 was considered as statistically significant.

## Results

### Phase 1: The development and validation of the student acceptance of Interprofessional learning (SAIL-10) instrument

A total of 203 students who participated in the prescribing skills workshop were requested to complete the SAIL-10 instrument. However, only 174 completed questionnaires were returned (response rate = 85.7%): medical students = 115 (66.1%); pharmacy students = 59 (33.9%). The majority of participants were 22 years old (97.7%) and female (67.3%).

#### Validity

Flesch reading ease of SAIL-10 was 44.9**.** Factor analysis showed that SAIL-10 consisted of two domains: “facilitators of interprofessional learning” (items no. 1–2, 5–6 and 8–10) and “acceptance to learning in groups” (items no. 3–4 and 7) [Table 1]. The KMO measure for sampling adequacy was 0.875, and the Bartlett’s test of sphericity produced an approximate chi-square of 883.713, *p* < 0.001, which explained 63.9% of the variance (“facilitators of interprofessional learning” = 42.7%; “acceptance to learning in groups” = 21.3%).

**Table 1:** Psychometric properties of the Student Acceptance of Interprofessional Learning (SAIL-10) instrument

No.	Item	Factor loadings		Overall Cronbach alpha	Cronbach alpha (by domain)	Corrected item-total correlation	Cronbach alpha if item deleted	Test-retest Spearman rho (*p*-value)**
		Domain 1	Domain 2					
6	The workshop strengthens the ties between the different healthcare professionals.	0.791		0.870	0.899	0.688	0.885	0.398 (*p* < 0.001)**
2	The presence of students from other healthcare professions in the workshop broadens the scope of my learning, knowledge and skills.	0.781				0.757	0.879	0.279 (*p* < 0.001)**
10	More interprofessional workshops should be conducted in undergraduate courses.	0.761				0.771	0.876	0.455 (*p* < 0.001)**
9	I recommend that inter-professional learning be incorporated into the curriculum.	0.760				0.731	0.881	0.242 (0.002)**
1	It is more interesting to be in a workshop with students from other healthcare professions than just my own course mates.	0.755				0.698	0.884	0.370 (*p* < 0.001)**
8	The discussion and feedback from students of other healthcare profession enrich my understanding of the workshop topic.	0.750				0.747	0.879	0.255 (0.001)**
5	I understand the roles of other healthcare professions well.	0.732				0.557	0.901^a^	0.311 (*p* < 0.001)**
4	I prefer to learn about prescribing skills among my own course mates only.		0.883		0.671	0.620	0.377	0.528 (*p* < 0.001)**
3	The presence of students from other healthcare professions in the workshop disrupts my learning ability.		0.772			0.496	0.599	0.273 (0.001)**
7	It is a waste of time to put students from different healthcare professions in the same workshop.		0.540			0.414	0.686^a^	0.306 (*p* < 0.001)**
	Total score							0.550 (*p* < 0.001)**

N.B. The word “workshop” in this instrument may be replaced by another word that appropriately describes the IPL intervention used

^a^Increase in Cronbach’s alpha value if item was deleted; **Statistically significant at *p* < 0.01

Domain 1 = Facilitators of inter-professional learning

Domain 2 = Acceptance to learning in groups

The total SAIL-10 score between medical and pharmacy students were significantly different (*p* = 0.038). When the two domains within the SAIL-10 was analyzed individually, the score for “acceptance to learning in groups” domain was significantly higher among medical students when compared to pharmacy students (*p* = 0.032). However, there was no significant difference in the score for “facilitators of IPL” domain (Table 2).

**Table 2:** Discriminative validity of SAIL-10

Domain	Name	Programme	No. of participants	Median SAIL score (IQR)	Mean rank	z-value^a^ (*p*-value)
Total score		Medical Pharmacy	115 59	84 (20) 80 (18)	93.18 76.43	−2.079 (0.038)*
1	Facilitators of inter-professional learning	Medical Pharmacy	115 59	28 (6) 27 (6)	92.59 77.76	−1.833 (0.067)
2	Acceptance to learning in groups	Medical Pharmacy	115 59	13 (4) 12 (4)	93.24 76.31	2.143 (0.032)*

^a^From Mann-Whitney U-test; *p < 0.05

#### Reliability

The Cronbach’s alpha value of the overall SAIL-10 instrument was 0.870. When each domain was analyzed separately, the Cronbach’s alpha value for “facilitators of IPL” and “acceptance to learning in groups” domains were 0.899 and 0.671, respectively. If item 5 was deleted, the Cronbach’s alpha value for the domain “facilitators of IPL” would increase slightly from 0.899 to 0.901. Similarly, if item 7 was deleted, the Cronbach alpha value for the domain “acceptance to learning in groups” would increase slightly from 0.671 to 0.686. All items had corrected item-total correlation values of more than 0.2 (Table 1).

At retest, only 157 out of 174 respondents returned the completed questionnaire (response rate = 89.7%). Spearman’s rho values for test-retest ranged from 0.242 to 0.528 (Table 1).

### Phase 2: Assessment of student acceptance of interprofessional learning

A total of 368 students attended the prescribing skills workshop, but only 270 students returned the completed questionnaire (response rate = 73.4%). The majority of respondents were 22 to 23 years of age (95%) and female (73.6%). There was no significant difference in the total SAIL-10 score, as well as for both domains, between medical and pharmacy students before attending the prescribing skills workshop. The total SAIL-10 score, as well as the scores for both domains were significantly higher after the participants attended the prescribing skills workshop (Table 3).

**Table 3:** Acceptance of interprofessional learning by medical and pharmacy students before and after a prescribing skills workshop

	Total no. of students	Total SAIL-10 score		Domain 1		Domain 2	
				Facilitators of inter-professional learning domain		Acceptance to learning in groups	
		Median (IQR)	Mean rank	Median (IQR)	Mean rank	Median (IQR)	Mean rank
Pre- prescribing skill workshop	270	72 (18)	81.66	25 (8)	89.58	12 (4)	96.67
Post- prescribing skill workshop	270	82 (22)	131.93	28 (7)	124.27	12 (5)	100.12
z-value^a^			−9.950		−10.310		−4.471
*p*-value			< 0.001**		< 0.001**		< 0.001**

a = From Wilcoxon signed ranks test

**Statistically significant at *p* < 0.01

Medical students showed significantly higher total SAIL-10 score, as well as for both domains, than pharmacy students after they completed the workshop (Table 4).

**Table 4:** Acceptance of interprofessional learning between medical and pharmacy students pre-post prescribing skill workshop

	Programme	No. of participants	Median SAIL score (IQR)	Mean rank	z-value^a^ (*p*-value)
Total SAIL-10 score					
Pre-prescribing skills workshop	Medical	175	72 (16)	136.83	−0.381
	Pharmacy	95	72 (16)	133.83	(0.703)
Post-prescribing skill workshop	Medical	175	84 (20)	146.75	−3.220
	Pharmacy	95	76 (22)	114.77	(0.001)**
Domain 1 score: Facilitators of interprofessional learning domain					
Pre-prescribing skills workshop	Medical	175	24 (7)	140.74	−0.814
	Pharmacy	95	25 (7)	132.66	(0.416)
Post-prescribing skill workshop	Medical	175	28 (8)	143.09	−2.186
	Pharmacy	95	28 (9)	121.51	(0.029)*
Domain 2 score: Acceptance to learning in groups					
Pre-prescribing skills workshop	Medical	175	12 (4)	142.15	−1.914
	Pharmacy	95	11 (4)	123.26	(0.056)
Post-prescribing skill workshop	Medical	175	13 (4)	145.87	−3.019
	Pharmacy	95	12 (5)	116.39	(0.003)**

^a^From Mann-Whitney U test;

*Statistically significant at *p* < 0.05; **p < 0.01

## Discussion

The SAIL-10 instrument was designed to assess student acceptance of IPL among medical and pharmacy students. It was developed using a systematic and rigorous process, according to standard guidelines for developing questionnaires [36]. The final version of SAIL-10 has 10 items. Flesch reading ease was 44.9. Exploratory factor analysis found that SAIL-10 has two domains: “facilitators of IPL” and “acceptance to learning in groups”. The overall Cronbach’s alpha was > 0.6 in each domain and this indicates adequate reliability.

Ideally, the SAIL-10 instrument should have a Flesch reading ease score of 60–70, so that it can be easily understood by students aged 12–13 years who are in the 7th grade in the United States [39]. Instead, the Flesch reading ease of the SAIL-10 instrument was 44.9. This means that our instrument is best understood by college graduates. Since SAIL-10 was specifically developed to assess the acceptance of IPL among university undergraduates, the Flesch reading ease was acceptable.

Our initial hypothesis was that the SAIL-10 instrument would be a 1-factor model, which was to assess the acceptance of medical and pharmacy students towards IPL. However, factor analysis showed that SAIL-10 consisted of two domains: “facilitators of IPL” (items no. 1–2, 5–6 and 8–10) and “acceptance to learning in groups” (items no. 3–4 and 7). A search of published literature on IPL found that there are three areas which would facilitate IPL: tactics (commencing IPL early in the programme, ensuring that IPL is valued, meeting students’ short-term learning needs), teamwork (meeting the challenges, involving committed people, employing a facilitator, developing your team, and discouraging individualism), and talk (improving the learning environment, increasing student interaction, improving group dynamics, embracing and resolving conflict) [40]. In contrast, according to a meta-analysis of 241 studies on the effects of the presence of other people on human task performance, it was found that the presence of others may impair complex performance accuracy and learning of a person in the group [35]. Therefore, it was not surprising that SAIL-10 yielded two domains.

The total SAIL-10 score between medical and pharmacy students were significantly different (*p* = 0.038), indicating that SAIL-10 was able to discriminate between medical and pharmacy students.

The Cronbach’s alpha value of the overall SAIL-10 instrument was 0.870 and for the two domains were 0.899 and 0.671, indicating that the items in the SAIL-10 instrument have adequate internal consistency. In test-retest, all of the items in SAIL-10 had fair to moderate/good correlation. This indicates that the SAIL-10 has stable reliability.

The SAIL-10 total score as well as the scores for both domains were significantly higher after participants attended the prescribing skills workshop. This implies that both the medical and pharmacy students accepted IPL better after undergoing the workshop. The content of the prescribing skills workshop was based on the World Health Organisation (WHO) Guide to Good Prescribing [34], which caters more for medical students. This was reflected by the medical students having significantly higher total SAIL-10 score, compared to pharmacy students. Medical and pharmacy students were equally distributed among the 6 expert stations (each has about 5 or 6 medical and 2 or 3 pharmacy students) that formed the “Jigsaw” technique for prescribing skills. These included: (i) defining your patient’s problem and specifying your therapeutic objective; (ii) selecting P-drugs for your own formulary; (iii) verifying the suitability of your P-drugs; (iv) sources of drug information and drug costing; (v) prescription writing and dosage calculation; (vi) giving information, instruction and warning; and monitoring or stopping treatment [34].

Findings of this study suggest that pharmacy students were less enthusiastic about the IPL workshop than medical students, as the steps involved in this workshop followed the prescribing process, which were perceived as a medical doctor’s role. In contrast, another study in Malaysia, which measured the attitude of medical, pharmacy and nursing students in a public medical school towards IPL, found that pharmacy and nursing students were significantly more willing to be engaged in IPL compared to medical students [24]. However, in this earlier study, the participants answered the RIPLS instrument without actually interacting with students from other disciplines [24]. Therefore, there is a possibility that their perceptions could change if they are to engage in an IPL programme, as reported by another study [41].

The main strength of the present study is that an instrument (i.e. SAIL-10) has been developed and validated to assess the acceptance of students towards IPL activities. In addition, the SAIL-10 instrument was tested on two separate cohorts of students and verified that the prescribing skills workshop conducted was an acceptable IPL activity. One of the limitations of the present study was that convergent validity could not be performed as there was no other instrument available to assess IPL, which has been validated in Malaysia. In addition, the Flesch reading ease is usually calculated for native English speakers. However, since the SAIL-10 was only meant for those in tertiary education, it was postulated that it should also be easily understood by medical and pharmacy students (who would have completed tertiary entry requirements). Another limitation of the study was that it recruited participants from one university only. This limits the generalisability of the results. In addition, approximately 75% of the participants were female, but this reflects the actual ratio of male to female students in Malaysian public universities [24]. One of the main limitations of this study is that the SAIL-10 has been tested in only one type of interprofessional activity (the prescribing skills workshop). However, the term ‘workshop’ in the SAIL-10 is a generic term and can be substituted with other interprofessional activities, such as research project or assignment, and be used to assess such activity.

## Conclusions

The English version of the SAIL-10 instrument, which consists of 10 items in 2 domains, is a valid and reliable instrument for assessing the acceptance of IPL between medical and pharmacy students in Malaysia. Future studies should include a larger cohort of students and to use different interprofessional activities to provide more valuable insights into the potential challenges in conducting IPL in local institutions. In addition, the workshop could be modified slightly to engage the pharmacy students more so that the IPL can be optimized for both groups of healthcare students.

## Abbreviations

IEPS: Interdisciplinary Education Perception Scale
IPL: Interprofessional learning
RIPLS: Readiness for Inter-Professional Learning Scale
SAIL: Student Acceptance of Interprofessional Learning
WHO: World Health Organisation

## References

[1] Darlow B, Coleman K, McKinlay E, Donovan S, Beckingsale L, Gray B (2015). The positive impact of interprofessional education: a controlled trial to evaluate a programme for health professional students. Bmc Med Educ..

[2] Lapkin S, Levett-Jones T, Gilligan C (2013). A systematic review of the effectiveness of interprofessional education in health professional programs. Nurs Educ Today..

[3] McKinnon A, Jorgenson D (2009). Pharmacist and physician collaborative prescribing: for medication renewals within a primary health Centre. Can Fam Physician.

[4] Leonard M, Graham S, Bonacum D (2004). The human factor: the critical importance of effective teamwork and communication in providing safe care. Quality & safety in health care.

[5] Gilligan C, Outram S, Levett-Jones T (2014). Recommendations from recent graduates in medicine, nursing and pharmacy on improving interprofessional education in university programs: a qualitative study. Bmc Med Educ.

[6] Hughes CM, McCann S (2003). Perceived interprofessional barriers between community pharmacists and general practitioners: a qualitative assessment. Br J Gen Pract.

[7] Cote L, Normandeau M, Maheux B, Authier L, Lefort L (2013). Collaboration between family physicians and community pharmacists opinions of graduates in family medicine. Can Fam Physician.

[8] World Health Organization W (2010). Framework for action on Interprofessional education and collaborative practice.

[9] Parsell G, Spalding R, Bligh J (1998). Shared goals, shared learning: evaluation of a multiprofessional course for undergraduate students. Med Educ.

[10] Finch J (2000). Interprofessional education and teamworking: a view from the education providers. BMJ..

[11] Cooper H, Carlisle C, Gibbs T, Watkins C (2001). Developing an evidence base for interdisciplinary learning: a systematic review. J Adv Nurs.

[12] Leaviss J (2000). Exploring the perceived effect of an undergraduate multiprofessional educational intervention. Med Educ.

[13] Parsell G, Bligh J (1999). The development of a questionnaire to assess the readiness of health care students for interprofessional learning (RIPLS). Med Educ.

[14] Coster S, Norman I, Murrells T, Kitchen S, Meerabeau E, Sooboodoo E (2008). Interprofessional attitudes amongst undergraduate students in the health professions: a longitudinal questionnaire survey. Int J Nurs Stud.

[15] Curran VR, Deacon DR, Fleet L (2005). Academic administrators’ attitudes towards interprofessional education in Canadian schools of health professional education. J Interprof Care.

[16] Gilbert JH (2005). Interprofessional education for collaborative, patient-centred practice. Nurs Leadersh (Tor Ont).

[17] Horsburgh M, Merry AF, Seddon M (2005). Patient safety in an interprofessional learning environment. Med Educ.

[18] Luecht RM, Madsen MK, Taugher MP, Petterson BJ (1990). Assessing professional perceptions: design and validation of an interdisciplinary education perception scale. J Allied Health.

[19] Lindqvist S, Duncan A, Shepstone L, Watts F, Pearce S (2005). Case-based learning in cross-professional groups - the development of a pre-registration interprofessional learning programme. J Interprof Care..

[20] Horsburgh M, Lamdin R, Williamson E (2001). Multiprofessional learning: the attitudes of medical, nursing and pharmacy students to shared learning. Med Educ.

[21] Baxter SK (2004). Perspectives and practice: speech and language therapy student views of an interprofessional learning experience. Learn Health Soc Care.

[22] Hind M, Norman I, Cooper S, Gill E, Hilton R, Judd P (2003). Interprofessional perceptions of health care students. J Interprof Care..

[23] Morison S, Boohan M, Moutray M, Jenkins J (2004). Developing pre-qualification inter-professional education for nursing and medical students: sampling student attitudes to guide development. Nurse Educ Pract.

[24] Aziz Z, Teck LC, Yen PY. The attitudes of medical, nursing and pharmacy students to inter-professional learning. Procd Soc Behv. 2011;29. 10.1016/j.sbspro.2011.11.287.

[25] Hawk C, Buckwalter K, Byrd L, Cigelman S, Dorfman L, Ferguson K (2002). Health professions students' perceptions of interprofessional relationships. Acad Med.

[26] Goelen G, De Clercq G, Huyghens L, Kerckhofs E (2006). Measuring the effect of interprofessional problem-based learning on the attitudes of undergraduate health care students. Med Educ.

[27] Mahler C, Berger S, Reeves S (2015). The readiness for Interprofessional learning scale (RIPLS): a problematic evaluative scale for the interprofessional field. J Interprof Care..

[28] Reeves S, Freeth D, McCrorie P, Perry D (2002). 'It teaches you what to expect in future . . . ': interprofessional learning on a training ward for medical, nursing, occupational therapy and physiotherapy students. Med Educ.

[29] Bowling A (2009). Research methods in health: investigating health and health services.

[30] Munro BH (2005). Statistical methods for health care research.

[31] Schwab DP, LLC BMS (1980). Construct validity in organization behavior. Research in organizational behavior.

[32] Aronson E (1978). The jigsaw classroom.

[33] Sim SM, Foong CC, Tan CH, Lai PS, Chua SS, Mohazmi M (2014). The use of jigsaw learning technique in teaching medical students prescribing skills. Med Teach.

[34] de Vries TPGM, Henning RH, Hogerzeil HV, Fresle DA. Guide to good prescribing: A practical manual. Geneva: World Health Organisation; 1995. http://apps.who.int/medicinedocs/pdf/whozip23e/whozip23e.pdf. Accessed 1 Mar 2017.

[35] Bond CF, Titus LJ (1983). Social facilitation: a meta-analysis of 241 studies. Psychol Bull.

[36] Campbell SM, Braspenning J, Hutchinson A, Marshall MN (2003). Research methods used in developing and applying quality indicators in primary care. BMJ..

[37] Sushil S, Verma N (2010). Questionnaire validation made easy. Eur J Scientific Res.

[38] Cohen J (1988). Statistical power analysis for the behavioral sciences.

[39] Kincaid JP, Fishburne J, Robert P, Rogers RL, Chissom BS (1975). Derivation of new readability formulas (automated readability index, fog count and Flesch Reading ease formula) for navy enlisted personnel.

[40] Begley CM (2008). Developing inter-professional learning: tactics, teamwork and talk. Nurs Educ Today.

[41] Taylor D, Yuen S, Hunt L, Emond A (2012). An interprofessional pediatric prescribing workshop. Am J Pharm Educ.

